# Underutilization of Pre-Exposure Prophylaxis Services Among Transgender and Nonbinary Youth: Findings from Project Moxie and TechStep

**DOI:** 10.1089/trgh.2019.0027

**Published:** 2019-10-01

**Authors:** Keith J. Horvath, Kieran Todd, Sean Arayasirikul, Nicholas W. Cotta, Rob Stephenson

**Affiliations:** ^1^ Department of Psychology, San Diego State University, San Diego, California.; ^2^ Division of Epidemiology and Community Health, University of Minnesota, Minneapolis, Minnesota.; ^3^ Center for Sexuality and Health Disparities, University of Michigan School of Nursing, University of Michigan, Ann Arbor, Michigan.; ^4^ Department of Pediatrics, University of California San Francisco, San Francisco, California.; ^5^ Division of Epidemiology and Community Health, University of Minnesota, Minneapolis, Minnesota.; ^6^ Department of Systems, Population and Leadership and Center for Sexuality and Health Disparities, University of Michigan, Ann Arbor, Michigan.

**Keywords:** adolescents, barriers to care, HIV/AIDS, PrEP, prevention, transgender

## Abstract

We use quantitative and qualitative data from two ongoing studies to describe pre-exposure prophylaxis (PrEP) awareness, willingness to use PrEP, barriers to facilitators of PrEP uptake, and PrEP use among 15- to 24-year-old transgender and gender nonbinary (TGNB) youth. Most youth were aware of PrEP, but only one participant across both studies reported current use. Uncertainty about willingness to take PrEP may be related to general (e.g., medication cost) and trans-specific (e.g., PrEP–hormone interactions) concerns. Intensified and sustained efforts are needed to engage TGNB youth along the PrEP continuum and the impact of new PrEP administration and dosing options should be examined for TGNB youth.

## Background

Pre-exposure prophylaxis (PrEP) has been shown to be efficacious for reducing the risk of HIV transmission by >90% if taken as prescribed.^1,2^ Despite the known efficacy of PrEP, recent studies show that transgender (trans) and gender nonbinary (gnb) (TGNB) youth have suboptimal PrEP awareness and very limited uptake.^3^ One study found that 62% of 18- to 29-year-old transgender women met the Centers for Disease Control and Prevention's PrEP needs threshold, yet only 31% knew about PrEP.^4^ However, transgender women who receive information about PrEP report high levels of willingness to use it.^5^ A recent study of >400 transgender men showed that one-quarter were eligible for PrEP, and only one-third had received information about PrEP from their provider.^6^ Although PrEP is now approved for adults and minors, little is known of engagement in the PrEP cascade among TGNB youth and young adults. This report uses quantitative and qualitative data from two ongoing studies to describe PrEP awareness, willingness to use PrEP, barriers to facilitators of PrEP uptake, and PrEP use among 15- to 24-year-old TGNB youth.

## Methods

### Data sources

Project Moxie is a feasibility study of an at-home HIV testing intervention for US TGNB youth (ages 15–24 years) recruited through social media.^7^ TGNB youth were recruited between June 2017 and June 2018, using advertisements and postings placed on social media sites. A total of 202 individuals took the online baseline survey and answered questions about their knowledge of PrEP, willingness to use PrEP, and their preferred modality for using PrEP (e.g., daily oral medication, injectable PrEP). Quantitative data from Project Moxie were used to describe levels of knowledge of PrEP and willingness to use PreP.

TechStep is a three-arm, randomized controlled trial, with a stepped care approach, for reducing sexual risk behaviors (e.g., condomless anal intercourse) and increasing PrEP uptake. To inform intervention development, four focus groups (*n*=34 participants) were held, during which TGNB youth (ages 15–24 years) were asked about their knowledge, attitudes, and experiences of PrEP, and about barriers to PrEP adherence and engagement in PrEP care. Participants were recruited by partnering clinics in Los Angeles and Houston, which used community outreach and referrals from local trans youth organizations to recruit and enroll participants. Qualitative data from TechStep were used to describe knowledge of PrEP and barriers and facilitators to PrEP use.

All aspects of this study were approved of by the University of Michigan and the University of North Carolina institutional review boards.

### Data analysis

Quantitative analysis examined differences in PrEP awareness, use of PrEP, willingness to use PrEP, and preferred modality of PrEP usage among four categories of gender identity: transfeminine, transmasculine, nonbinary assigned female at birth, and nonbinary assigned male at birth. Not all 202 participants answered all questions, resulting in some missing data. In addition, because of a survey programming error, data on willingness to use PrEP were only collected for 77 participants. Nonparametric tests of proportion (chi-square and Fisher's exact) were used to assess group differences in outcomes by gender identity, using a statistical significance level (α) of 0.05. Analyses were conducted using Stata Version 12.0 (Stata Corp.)

Focus groups were audio-recorded and transcribed verbatim. Qualitative data analysis was conducted by trained research staff using a conventional approach to content analysis.^8–10^ Two separate staff reviewed and coded transcripts to identify emergent themes and categories related to PrEP among trans/gnb youth. Codes were identified inductively from the data rather than conceived *a priori*.^9^ Codes were then labeled and organized into categories, building on the emergent relationship between codes and categories.^9^ The final coding scheme was a product of an iterative process involving input from three research staff and discussions to resolve disagreements.

## Results

Across Project Moxie's study sample of 202 TGNB youth, 66.8% of participants identified as non-Hispanic white, 19.3% were unemployed, and not a student, and 37.6% identified their sexual orientation as queer. As given in Table 1, 56.1% (*n*=106) of 189 respondents reported hearing of PrEP, and 0.5% (*n*=1) were currently using PrEP (13 respondents did not answer this question). In addition, 52.0% (*n*=40) of 77 respondents when asked about willingness to use PrEP said they were willing, 9.0% (*n*=7) were not willing to use PrEP, and 39.0% (*n*=30) were unsure of their willingness. There were no significant differences among the four gender categories on their willingness to use PrEP; however, a higher proportion of transmasculine participants were willing to use PrEP than transfeminine participants (*p*=0.014). Those who reported a willingness to use PrEP were asked to indicate which of the following methods of taking PrEP they would most prefer: taking one pill every day (i.e., the currently approved PrEP dosing method; *n*=51, 26.8%), taking one pill before having sex (*n*=62, 32.6%), injecting PrEP every 3–4 months (*n*=66, 34.7%), and applying a lubricant before and after having anal sex (*n*=11, 5.8%). Altogether, nearly two-thirds of participants (73.2%; *n*=139) indicated their preferred method of using PrEP that was not the once-a-day pill.

**Table 1.** Measures of Engagement in Pre-Exposure Prophylaxis Care in Transgender Youth, United States, June 2017–June 2018

	*Total (*n*=189),* n *(%)*	*Transfeminine (*n*=33),* n *(%)*	*Transmasculine (*n*=77),* n *(%)*	*Nonbinary AFAB (*n*=54),* n *(%)*	*Nonbinary AMAB (*n*=25),* n *(%)*
Heard of PrEP					
Yes	106 (56.1)	15 (60.0)	48 (62.3)	28 (51.9)	15 (45.5)
No	83 (43.9)	18 (40.0)	29 (37.7)	26 (48.2)	10 (40.0)
Using PrEP24					
Yes	1 (0.5)	0 (0.0)	1 (1.3)	0 (0.0)	0 (0.0)
No	183 (99.5)	32 (100.0)	74 (98.7)	53 (100.0)	24 (100.0)
Willing to use PrEP10					
Yes	40 (52.0)	5 (29.4)	18 (72.0)	14 (56.0)	3 (30.0)
No	7 (9.0)	2 (11.8)	2 (8.0)	2 (8.0)	1 (10.0)
I don't know	30 (39.0)	10 (58.9)	5 (20.0)	9 (36.0)	6 (60.0)
Preferred modality					
Approved method (once-a-day pill)	51 (26.8)	9 (27.3)	18 (23.1)	16 (29.6)	8 (32.0)
Unapproved methods	139 (73.2)	24 (72.7)	60 (76.9)	38 (70.4)	17 (68.0)

Taking one pill before sex, getting an injection every 3–4 months, or putting lubrication in anus (and/or partners anus) both before and after having anal sex.

AFAB, assigned female at birth; AMAB, assigned male at birth.

The themes and their related example quotes from TechStep focus group participants (mean age=21 years [range 17–24]; 50% white; 50% Latinx; 44% trans man, 28% trans woman or woman, 28% gnb; 56% unemployed; 44% in school; 66% temporary or unstable housing) are given in Table 2, and described hereunder.

**Table 2.** Pre-Exposure Prophylaxis-Related Themes from TechStep Focus Groups with Transgender/Gender Nonbinary Youth (Ages 15–24 Years)

*Theme*	*Definition*	*Example quotes*
Awareness	Mentions of participants' awareness of PrEP	“I think it's really interesting that I haven't heard about it because you would think so many people would make this a well-known thing… so many people are afraid to talk about HIV and stuff, because so many people have that stigma that if you have this you're dirty… and it just needs more exposure.”
Knowledge	Mentions of participants' knowledge of PrEP	“A morning after like—I don't know if you take it before you have sex or after you have sex, but I know it's one of those… And it's supposed to stop any disease that will come your way.”
		“I wasn't sure if it was every day or not.”
		“I think you take it like every three months or something like that, I think.”
		“Do I like go to my endo or do I go to my general care. Do I tell my endo?”
Perceptions about and interest in taking PrEP	Mentions of participants' attitudes about, and interest in, PrEP	“It was just like I use protection almost like all the time. It's just how I practice. So, I was—that was just kind of like enough for me to not take it.”
		“I think just the worry of the fact that it is still new. That, like, it could be doing something to my body that I don't have any clue that it's doing to my body. Simply because like it's still on study. It's still being worked out.”
		“So, like, what further am I doing especially as someone's on hormones already and on like muscle relaxers and like on—because I had to take like Oxy multiple times for like different intense pains, and then, the fact that PrEP doesn't go well with some pain medications.”
Access and utilization	Mentions of participants' beliefs and experiences accessing and using PrEP	“It's the biggest thing or just having time to go to that provider to get checked every three months it's the biggest thing.”
		“I don't like doctors and I don't like pills.”
		“I've been taking medication since like I don't know, third grade probably and like I still forget to take my pills sometimes things come up you're like forget like that.”
		“Then also like getting to doctor's visits and I'm on my parents' insurance too like having to explain to them that I'm like sexually active and like I want to take it because like they get all the bills and stuff like that. So all that would also be complicated for that too.”

PrEP, pre-exposure prophylaxis.

1. PrEP awareness: There was a high level of awareness of PrEP among TechStep participants. Only one participant reported being unaware of PrEP before the focus group. Youth identified that HIV-related stigma may be a cause of the lack of awareness around PrEP. Participants heard about PrEP through community-based health care providers, research studies, and college classes.
2. PrEP knowledge: Many participants expressed little knowledge about PrEP. Specific topics of interest in need of more education included: dosing regimens (e.g., daily or event driven), the differences between PEP and PrEP, and drug-related saturation in the anus compared with the vagina. Participants were also unaware of how to access PrEP, specifically whether or not a prescription was necessary and how much PrEP costs.
3. Perceptions and interest in taking PrEP: Most participants perceived their own level of HIV risk as low and, therefore, were not interested in taking PrEP. Some reasons for having a low-risk perception included the following: consistent condom use, regular testing, abstinence, knowing their partner's status, and not having multiple partners. Other participants were less interested in taking PrEP because of potential concerns about their health, including potential contraindications with concomitant medications (e.g., hormones), the combined impact of multiple medications on their bodies, and the unknown long-term consequences of taking PrEP.
4. PrEP access and utilization: There was a low level of access and utilization of PrEP among participants in this sample. Barriers to PrEP utilization included cost, previous negative experiences of medical institutions, medical mistrust, concerns about disclosure while being covered through their parent's insurance. A few participants described having to engage with the medical system on a more frequent, regular basis as a part of routine PrEP-related care as a barrier to taking PrEP. Trans/gnb youth stated that they would be motivated to access PrEP services through a research protocol and by having access to a trusted and knowledgeable clinician. Participants also expressed interest in ways of accessing PrEP without a prescription to possibly circumvent unintended disclosures to parents and in injectable forms of PrEP to address barriers to adherence.

## Discussion

Little is known of engagement in the PrEP continuum among TGNB youth, a gap that we address in this study. The majority of youth in both the survey and the focus groups were aware of PrEP; however, only one person reported taking PrEP across both studies. The low uptake of PrEP mirrors previous studies that demonstrate low PrEP use among transgender youth overall.^11^ Although over half of the Project Moxie participants were willing to take PrEP, which was particularly evident among transgender men, a sizable proportion (39%) were not sure if they would be willing to take PrEP. In contrast, nearly all (33/34) participants in TechStep indicated an awareness of PrEP. We attribute this to the nature of each study. Project Moxie was conducted online, and may have reached trans youth who have less PrEP knowledge; TechStep was conducted in person and may have reached trans youth who were more likely to have been exposed to information about PrEP. The most prominent concerns about taking PrEP from the TechStep focus group participants included the cost of the medication and privacy and stigma-related concerns. Similar concerns were shared by transgender youth and young adults during qualitative interviews in a previous study.^12^ In addition, many of the youth in the focus groups were on their parent's insurance and were concerned about how to access PrEP without their parents knowing. Finally, some youth raised worries about how PrEP might interact with hormone therapy or other medications they were taking.

Limitations of these studies are that the results may not be representative of all TGNB youth because of the convenience sample that was drawn for both studies and the small sample size for the TechStep focus groups. In addition, missing data in Project Moxie related to willingness to use PrEP may have impacted study results. Our analysis did not account for differences in risk for HIV or PrEP eligibility in the analysis of differences in the PrEP outcomes among the gender groups. Transgender women have been shown to be at high risk for HIV, but a recent analysis showed that approximately one-quarter of transgender men meet PrEP eligibility requirements.^6,13^ Future studies assessing differences in PrEP outcomes between gender groups would benefit from the consideration of HIV risk and PrEP eligibility. In addition, the literature suggests that there is an increased risk for HIV among trans youth of color.^11,14^ We are limited in our understanding of how these youth engage with PrEP, and identify this as a priority for future research.

With these limitations in mind, these and previous results suggest that intensified and sustained efforts to increase awareness, willingness to use, and uptake of PrEP among transgender youth are critically needed. The low use of PrEP across both samples may be the result of transgender youth not believing that they are good candidates for PrEP, along with general (e.g., costs) and trans-specific (e.g., interaction with hormone therapy) barriers to engagement along the PrEP continuum. Such engagement may improve as new PrEP administration and dosing options (e.g., injectable PrEP; on-demand PrEP) become available, as the majority of trans/gnb youth in Project Moxie reported a preference for PrEP options other than daily oral medications.

## Abbreviations Used

AFAB: nonbinary assigned female at birth
gnb: gender nonbinary
PrEP: pre-exposure prophylaxis
TGNB: transgender and gender nonbinary
trans: transgender
